# Analytic and Unambiguous Phase-Based Algorithm for 3-D Localization of a Single Source with Uniform Circular Array

**DOI:** 10.3390/s18020484

**Published:** 2018-02-06

**Authors:** Le Zuo, Jin Pan, Boyuan Ma

**Affiliations:** 1 Department of Microwave Engineering, University of Electronic Science and Technology of China, Chengdu 611731, China; panjin@uestc.edu.cn (J.P.); 201621020208@std.uestc.edu.cn (B.M.); 2 School of Electrical and Electronic Engineering, Nanyang Technological University, 50 Nanyang Avenue, Singapore 639798, Singapore

**Keywords:** three-dimensional (3-D) localization estimation, uniform circular array (UCA), phase ambiguity, Fourier transform

## Abstract

This paper presents an analytic algorithm for estimating three-dimensional (3-D) localization of a single source with uniform circular array (UCA) interferometers. Fourier transforms are exploited to expand the phase distribution of a single source and the localization problem is reformulated as an equivalent spectrum manipulation problem. The 3-D parameters are decoupled to different spectrums in the Fourier domain. Algebraic relations are established between the 3-D localization parameters and the Fourier spectrums. Fourier sampling theorem ensures that the minimum element number for 3-D localization of a single source with a UCA is five. Accuracy analysis provides mathematical insights into the 3-D localization algorithm that larger number of elements gives higher estimation accuracy. In addition, the phase-based high-order difference invariance (HODI) property of a UCA is found and exploited to realize phase range compression. Following phase range compression, ambiguity resolution is addressed by the HODI of a UCA. A major advantage of the algorithm is that the ambiguity resolution and 3-D localization estimation are both analytic and are processed simultaneously, hence computationally efficient. Numerical simulations and experimental results are provided to verify the effectiveness of the proposed 3-D localization algorithm.

## 1. Introduction

Source localization using an array of sensors is an important topic for wireless communication, radar, and sonar, etc. For the problem of source localization, a uniform circular array (UCA) is attractive, as it provides two-dimensional (2-D) direction of arrival (DOA), 360° azimuth coverage, an almost identical beamwidth, and additional elevation angle information [1]. Recently, employing the characteristics of spherical wavefronts of near-field, a large number of publications were reported on applications of UCAs to three-dimensional (3-D) localization, where the range of the source is also obtained in addition to 2-D DOA [1,2,3,4,5]. Since either received phases or received complex voltages contain information about the wavefronts, both of them have been utilized in near-field 3-D localization. While complex-voltage-based 3-D MUSIC methods [2,3] have been widely studied, they require eigenvalue decomposition and expensive 3-D search procedure. Based on phases of the wavefronts, some closed-form algorithms were proposed via a least square estimation (LSE) [1,4,5]. Chen et al. [5] proposed a method for 3-D localization of a single source with a UCA. However, the number of sensors in [5] is restricted to being even and not less than eight. Jung [1] proposed a phase-based algorithm applicable to any number of sensors with a UCA. However, the LSE still involves matrix computation, which burdens the computational complexity. Furthermore, it is well known that high estimation accuracy can be obtained from large apertures. However, the measurement of phase difference can only be made modulo of 2π, which leads to an ambiguity in determining localization parameters of the source [6,7]. To solve phase ambiguity, a modulo conversion method [8] was proposed, but it is inherently developed for linear array interferometers and cannot be directly applied to a UCA, as a UCA’s phase differences are dependent on both elevation and azimuth angles. In addition, rotary ways were also used for ambiguity resolution of a UCA [9,10,11]. However, rotary interferometers face the problem of source correspondence and real-time applications. In [5], a subarray grouping and ambiguity searching method was proposed and the rough angle estimation was achieved by searching the nearest value among subarrays. However, a search procedure is needed, and the sensors must be in pairs.

Alternatively, in this paper, we propose an analytic and unambiguous phase-based algorithm for 3-D localization of a single source with a UCA. In order to sample the phases on a circular aperture, a signal model for phase extraction is first established with equivalent phase noises through observations of signal samples corrupted by an additive Gaussian white noise (AWGN). A localization algorithm is then developed by exploiting the centro-symmetry and periodicity of a UCA in the Fourier domain. The solutions to 3-D localization parameters are explicit Fourier transforms of phase distribution on the circumference of a circular aperture. Fourier sampling theorem ensures that five is the minimum element number for 3-D localization of a single source with a UCA. Moreover, accuracy analysis is addressed in the Fourier domain to provide mathematical insights into the proposed algorithm. Furthermore, the high-order difference invariance (HODI) property of a UCA is addressed to compress the phase range and thereby realizing ambiguity resolution. The advantage of the algorithm is that ambiguity resolution and localization estimation are both explicit and are processed simultaneously. Therefore, the proposed algorithm is computationally simple and efficient. Finally, numerical simulations and experimental measurements are provided to verify the effectiveness of the proposed algorithm.

This paper contributes to the area of 3-D localization estimation in the following aspects:

1. The estimation algorithm sufficiently exploits the centro-symmetry and periodicity of a circular aperture by Fourier transforms and has established algebraic relations between 3-D localization parameters and phase samples on the circumference of a UCA.
2. The phase-based HODI property of a UCA has been exploited, based on which a novel ambiguity resolution has been addressed without accuracy loss. Ambiguity resolution and parameter estimation have been merged into one process, resulting in a computationally efficient algorithm.
3. It is first revealed that the minimum number of sensors for single source localization with a UCA is five.
4. Estimation accuracy has been analyzed in the Fourier domain to provide mathematical insights into the parameter estimation using a UCA.

The rest of the paper is organized as follows. Section 2 establishes the signal model of 3-D localization of a single source. In Section 3, Fourier transforms are applied to a continuous circular aperture and discrete phase samples, leading to an analytic 3-D localization algorithm. Accuracy analysis is addressed in Section 4. In Section 5, the HODI of a UCA is found and ambiguity resolution is developed by compressed phase range. Numerical simulations and experimental measurements are provided to verify the proposed algorithm in Section 6 and Section 7, respectively. Section 8 concludes this paper. 

## 2. Signal Modeling

In this section, the periodic phase distribution on the circumference of a continuous circular aperture is first derived. Then in order to extract phases from sensor responses, a signal model is established. 

### 2.1. Phase Distribution

**Figure 1:**
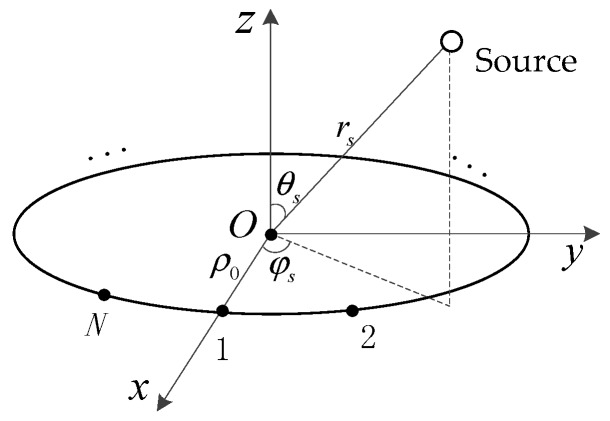
Geometry of a uniform circular array (UCA) with a single source.

Consider a sensor placed at $\left(\rho_{0},\pi /2,\varphi \right)$ in a spherical coordinate of (r,θ,φ), which is adopted for estimation of 3-D localization of a single source, where the range r is measured from the origin, the azimuth angle φ∈[0,2π) is measured counterclockwise from the *x* axis, and the elevation angle θ∈[0,π) is measured down from the *z* axis, as shown in Figure 1. For an emitting source located at $\left(r_{s},\theta_{s},\varphi_{s}\right)$ in the same spherical coordinate, the distance between the source and the sensor is given by [1]

(1) $$d={\left[\rho_{0}^{2}+r_{s}^{2}-2\rho_{0}r_{s}\sin\theta_{s}\cos\left(\varphi -\varphi_{s}\right)\right]}^{1/2}$$

Approximation of (1) by second-order Taylor series expansion leads to

(2) $$d≅r_{s}-\rho_{0}\sin\theta_{s}\cos\left(\varphi -\varphi_{s}\right)+\rho_{0}^{2}/\left(2r_{s}\right)\left[1-\sin^{2}\theta_{s}\cos^{2}\left(\varphi -\varphi_{s}\right)\right]$$

Then the phase distribution of the incident wave is expressed as

(3) $$\Phi \left(\varphi \right)=kr_{s}-k\left[\rho_{0}\sin\theta_{s}\cos\left(\varphi -\varphi_{s}\right)+\rho_{0}^{2}/\left(2r_{s}\right)\left(1-\sin^{2}\theta_{s}\cos^{2}\left(\varphi -\varphi_{s}\right)\right)\right]+\Phi_{int}$$

where k=2π/λ is the wave number, λ is the wavelength of the incident signal, and $\Phi_{int}$ is a constant representing the initial phase of the signal, which can be interpreted as the phase of the signal arriving at the center of the array.

A UCA can be deemed as *N* equally placed identical sensors that sample the circular aperture, i.e., the noiseless unambiguous phases of outputs of sensors are denoted by a discrete version of (3), namely

(4) $$\Phi_{i}=kr_{s}-k\left[\rho_{0}\sin\theta_{s}\cos\left(\varphi_{i}-\varphi_{s}\right)+\rho_{0}^{2}/\left(2r_{s}\right)\left(1-\sin^{2}\theta_{s}\cos^{2}\left(\varphi_{i}-\varphi_{s}\right)\right)\right]+\Phi_{int}$$

3-D localization is implemented by the acquisition of $\Phi_{i}$ across the circular aperture and then by the solution to its associated equation that relates the phase acquisition to the unknown parameters.

### 2.2. Phase Extraction

The response observed at a sensor output is the exponent of phase distribution corrupted by an AWGN. Consider the sensor output has the form y(t)=Aexp(jωt+jΦ)+n(t), where $j=\sqrt{-1}$, ω is the angular frequency of the signal, *A* is the magnitude of the signal, and n(t) is an AWGN with zero mean and variance $\sigma_{n}^{2}$. The signal-to-noise ratio (SNR) is defined by $\mathrm{SNR}=A^{2}/\sigma_{n}^{2}$. The frequency of the signal is assumed to be accurately estimated using a number of well-known techniques [12]. The phase of each receiver output can be obtained by [13]

(5) $$\tilde{\Phi }=\arg\left(\left(1/M\right)\sum_{t=1}^{M}y\left(t\right)e^{-j\omega Tt}\right)$$

where *T* is the inverse of the constant sampling rate, *M* is the number of snapshots, and arg(*x*) is the argument of *x*. Since phase ambiguity resolution is delayed to Section 5, the unambiguous phase is considered here for the sake of simplicity. At moderately high SNR, the AWGN can be converted into an equivalent additive phase noise [14], i.e.,

(6) $$\tilde{\Phi }=\arg\left(y\right)=\Phi +\xi$$

where ξ is the phase noise of the receiver. The variance of ξ is given by

(7) $$\mathrm{var}\left(\xi \right)=1/\left(2M\mathrm{SNR}\right)=\sigma^{2}$$

which is also Gaussian [15]. Hence, the phase noise model reveals interconnections between an AWGN and the phase noise. The analysis above suggests that the sampling noises are Gaussian. Besides, since a typical implementation of a passive RF system involves a dedicated signal acquisition channel (receiver, analog-to-digital converter, et al.) for each sensor, when the distributed phases are sampled, the phase measurements are contaminated by noises, arising from the presence of receiver noises, hardware imperfections, front end noises, et al. [16]. Then, the phase samples can be written as ${\tilde{\Phi }}_{i}=\Phi_{i}+\varepsilon_{i}$, where $\varepsilon_{i}$ is the overall phase noise on the *i*th receiving channel, which is assumed to be Gaussian with zero mean, $\sigma^{2}$ variance, and statistically independent [17], i.e.,

(8) $$E\left(\varepsilon_{i}\varepsilon_{j}\right)=\sigma^{2}\delta \left(i-j\right)$$

where δ(i) is a kronecker delta function.

## 3. Proposed 3-D Localization Algorithm

This section first applies Fourier transforms to the periodic phase distribution on the circumference of a continuous circular aperture and then to discrete phase samples. Moreover, algebraic relations between 3-D position parameters and Fourier spectrums are established. Finally, the proposed algorithm is compared to a previous method [1].

### 3.1. Continuous Aperture Phase Distribution

Noticing that the phase distribution on the circumference of a circular aperture, i.e., Φ(φ), is a periodic function of φ, we apply Fourier transforms and obtain the spectrum of Φ(φ), namely

(9) $$c_{n}=\left(1/2\pi \right)\int_{0}^{2\pi }\Phi \left(\varphi \right)\exp\left(jn\varphi \right)d\varphi =c_{0}\delta \left(n\right)-c_{1}\delta \left(n-1\right)-c_{1}^{*}\delta \left(n+1\right)-c_{2}\delta \left(n-2\right)-c_{2}^{*}\delta \left(n+2\right)$$

where * denotes complex conjugate, and

(10) $$c_{0}=kr_{s}+k\rho_{0}^{2}/\left(2r_{s}\right)-k{\left(\rho_{0}\sin\theta_{s}\right)}^{2}/\left(4r_{s}\right)+\Phi_{int}$$

(11) $$c_{1}=k\rho_{0}\sin\theta_{s}\exp\left(j\varphi_{s}\right)/2$$

(12) $$c_{2}=k\rho_{0}^{2}\sin^{2}\theta_{s}\exp\left(j2\varphi_{s}\right)/\left(8r_{s}\right)$$

Hence, we get the dependence of 3-D localization parameters on these three spectrums, namely $c_{0}$, $c_{1}$, and $c_{2}$. Careful examination of (11) reveals that the elevation angle and the azimuth angle can be decoupled from the magnitude and the argument of a single spectrum, namely

(13) $${\hat{\theta }}_{s}=\sin^{-1}\left(2\left|c_{1}\right|/\left(k\rho_{0}\right)\right)$$

(14) $${\hat{\varphi }}_{s}=\arg\left(c_{1}\right)$$

Similarly, the range of the source can be manipulated from two spectrums, given by

(15) $${\hat{r}}_{s}=\left|c_{1}^{2}/\left(2kc_{2}\right)\right|$$

As seen from (13) to (15), 3-D localization parameters can be decoupled from the manipulations of two Fourier spectrums, i.e., the two angular parameters are contained in the first-order Fourier spectrum, while the range parameter is inversely proportional to the magnitude of the second-order Fourier spectrum.

### 3.2. Discrete Phase Samples

The discrete version of (9) can be expressed as discrete Fourier transforms (DFTs) of phase samples on the circumference of a circular aperture, namely

(16) $$d_{n}=\frac{1}{N}\sum_{i=1}^{N}\Phi_{i}\exp\left(jn\varphi_{i}\right)$$

Utilization of Fourier sampling theorem gives

(17) $$d_{n}=\sum_{l=-\infty }^{+\infty }c_{n-lN}$$

Careful examination of (9) reveals that $c_{n}$ is non-zero only when n=−2, −1, 0, 1, 2. Therefore, if N≥5, no matter the element number is even or odd, the values of $d_{0}$, $d_{1}$, and $d_{2}$ remain the same as $c_{0}$, $c_{1}$, and $c_{2}$, respectively, since spectrum aliasing is avoided. Hence, combining (11), (12), and (17) gives two DFT spectrums that are related to the localization parameters, respectively, namely

(18) $$d_{1}=\left(1/2\right)k\rho_{0}\sin\theta_{s}\exp\left(j\varphi_{s}\right)$$

(19) $$d_{2}=k\rho_{0}^{2}\sin^{2}\theta_{s}\exp\left(j2\varphi_{s}\right)/\left(8r_{s}\right)$$

Considering phase noises, the two DFT spectrums are calculated as

(20) $${\tilde{d}}_{1}=d_{1}+\delta d_{1}$$

(21) $${\tilde{d}}_{2}=d_{2}+\delta d_{2}$$

where

(22) $$\delta d_{1}=\frac{1}{N}\sum_{i=1}^{N}\varepsilon_{i}\exp\left(j\varphi_{i}\right)$$

(23) $$\delta d_{2}=\frac{1}{N}\sum_{i=1}^{N}\varepsilon_{i}\exp\left(j2\varphi_{i}\right)$$

are the first-order and the second-order DFT spectrums of phase noises, respectively. Neglecting the noises, algebraic formulations for angular parameter estimations are extracted from the magnitude and the argument of the first-order DFT spectrum, respectively, namely

(24) $${\tilde{\theta }}_{s}=\sin^{-1}\left(2\left|{\tilde{d}}_{1}\right|/\left(k\rho_{0}\right)\right)=\sin^{-1}\left(2\left|\sum_{i=1}^{N}{\tilde{\Phi }}_{i}\exp\left(j\varphi_{i}\right)\right|/\left(Nk\rho_{0}\right)\right)$$

(25) $${\tilde{\varphi }}_{s}=\arg\left({\tilde{d}}_{1}\right)=\arg\left(\sum_{i=1}^{N}{\tilde{\Phi }}_{i}\exp\left(j\varphi_{i}\right)\right)$$

Meanwhile, the estimation of range is manipulated by $d_{1}$ and $d_{2}$, and given by

(26) $${\tilde{r}}_{s}=\left|{\tilde{d}}_{1}^{2}/\left(2k{\tilde{d}}_{2}\right)\right|=\left|{\left(\sum_{i=1}^{N}{\tilde{\Phi }}_{i}\exp\left(j\varphi_{i}\right)\right)}^{2}/\left(2Nk\sum_{i=1}^{N}{\tilde{\Phi }}_{i}\exp\left(j2\varphi_{i}\right)\right)\right|$$

Equations (24)–(26) have established the algebraic relations between 3-D localization parameters and phase samples. The two angular parameters are decoupled to the first-order DFT spectrum and the range parameter is decoupled to the manipulation of the first-order and the second-order DFT spectrums. It is worth mentioning that these formulations are explicit and involve no matrix operations. Hence, the algorithm is analytic and computationally simple. It is also worth noting that the initial phase, i.e., $\Phi_{int}$, is immaterial, provided that it is the same for all the elements and does not appear in these estimation formulations. Alternatively and conveniently, the phases can be measured with respect to a particular array element. This is very useful for hardware implementation of UCA interferometers because a phase interferometer is a system based on phase differences, and $\Phi_{int}$ in (4) can be set to arbitrary one from $-\Phi_{1}$ to $-\Phi_{N}$, indicating that the phase differences can be measured from each sensor with reference to one arbitrary sensor and do not affect the solution to 3-D localization parameter estimation.

### 3.3. Equivalence to Previous Method

Observe that the LSE $\hat{b}={\left(A^{T}A\right)}^{-1}A^{T}\hat{u}$, i.e., (11) in [1], can be reformulated by *N* terms, instead of (*N* − *m*) terms, where *m* denotes *n* in [1], i.e., rewrite (10c) in [1] as

(27) $$A=\left[\begin{array}{cc} \cos\varphi_{1}-\cos\varphi_{1+m} & \sin\varphi_{1}-\sin\varphi_{1+m}\\ \begin{array}{l} \cos\varphi_{2}-\cos\varphi_{2+m}\\ \;\vdots \\ \cos\varphi_{N-m}-\cos\varphi_{N}\\ \;\vdots \\ \cos\varphi_{N}-\cos\varphi_{m} \end{array} & \begin{array}{l} \sin\varphi_{2}-\sin\varphi_{2+m}\\ \;\vdots \\ \sin\varphi_{N-m}-\sin\varphi_{N}\\ \;\vdots \\ \sin\varphi_{N}-\sin\varphi_{m} \end{array} \end{array}\begin{array}{cc} \cos\left(2\varphi_{1}\right)-\cos\left(2\varphi_{1+m}\right) & \sin\left(2\varphi_{1}\right)-\sin\left(2\varphi_{1+m}\right)\\ \begin{array}{l} \cos\left(2\varphi_{2}\right)-\cos\left(2\varphi_{2+m}\right)\\ \;\vdots \\ \cos\left(2\varphi_{N-m}\right)-\cos\left(2\varphi_{N}\right)\\ \;\vdots \\ \cos\left(2\varphi_{N}\right)-\cos\left(2\varphi_{m}\right) \end{array} & \begin{array}{l} \sin\left(2\varphi_{2}\right)-\sin\left(2\varphi_{2+m}\right)\\ \;\vdots \\ \sin\varphi_{N-m}-\sin\left(2\varphi_{N}\right)\\ \;\vdots \\ \sin\left(2\varphi_{N}\right)-\sin\left(2\varphi_{m}\right) \end{array} \end{array}\right]$$

and redefined (10a) in [1] as

(28) $$u=\left[u_{1,1+m}\;u_{2,2+m}\cdots u_{N-m,N}\cdots u_{N,m}\right]$$

Then (12a), (12b), and (12c) in [1] are the same as (24), (25), and (26), respectively. It is worth noting that if the number of terms in the LSE equals that of elements, the solutions to the LSE are the same, irrelevant to the choice of *m*, and equivalent to our proposed algorithm.

## 4. Accuracy Analysis

Note that the proposed algorithm exploits DFTs, and therefore its estimation accuracy should also be interpreted in the context of the Fourier domain. Accordingly, accuracies of the 3-D localization estimation are derived from accuracies of Fourier spectrums. 

### 4.1. Accuracy of Fourier Spectrums

Rewrite the DFTs of phase noises as

(29) $$\delta d_{n}=P_{n}+jQ_{n},n=1,2,\ldots ,N-1$$

According to (8) and (22), (23), $P_{n}$ and $Q_{n}$ both conform to the Gaussian distribution, with means of zero and variances of ${\left(2\pi \sigma \right)}^{2}/\left(2N\right)$. Additionally, based on the orthogonality of DFTs, different spectrums are mutually independent.

### 4.2. Accuracy of 3-D Localization Estimation

Accuracies of the 3-D localization estimation is affected by projection of noises upon DFT spectrums. First, accuracies of the angle estimations are analyzed. First-order approximation of the derivative of $d_{1}$ leads to

(30) $$\delta d_{1}=k\rho_{0}\left(e^{j\varphi_{s}}\cos\theta_{s}\delta \theta_{s}+j\sin\theta_{s}e^{j\varphi_{s}}\delta \varphi_{s}\right)/2$$

Comparisons of the real and imaginary parts of (29) with those of (30) yield that means of the two angular parameters are zero, and variances of the two angular parameters are given by

(31) $$\mathrm{var}\left(\delta \theta_{s}\right)=\frac{2\sigma^{2}}{N{\left(k\rho_{0}\cos\theta_{s}\right)}^{2}}$$

(32) $$\mathrm{var}\left(\delta \varphi_{s}\right)=\frac{2\sigma^{2}}{N{\left(k\rho_{0}\sin\theta_{s}\right)}^{2}}$$

Next, we investigate the accuracy of the range estimation by a UCA. First-order approximation of the derivative of ${\tilde{r}}_{s}$ yields

(33) $$\delta r_{s}=\frac{1}{2k}\left|\frac{8r_{s}\delta d_{1}}{\rho_{0}\sin\theta_{s}\exp\left(j\varphi_{s}\right)}-\frac{{\left(4r_{s}\right)}^{2}\delta d_{2}}{{\left(\rho_{0}\sin\theta_{s}\exp\left(j\varphi_{s}\right)\right)}^{2}}\right|$$

Substituting (29), (30) into (33), and after some manipulations, we obtain that the mean of the range estimation is zero, and the variance of the range estimations is given by

(34) $$\mathrm{var}\left(\delta r_{s}\right)={\left(\frac{4r_{s}}{k\rho_{0}\sin\theta_{s}}\right)}^{2}\left[1+{\left(\frac{2r_{s}}{\rho_{0}\sin\theta_{s}}\right)}^{2}\right]\frac{\sigma^{2}}{2N}$$

As seen from (31), (32), and (34), the variances of parameter estimations with a UCA are independent of the azimuth angle, which agrees with the rotation invariance of circular arrays. Meanwhile, larger number of elements gives lower variances of 3-D position parameters, and hence gives higher estimation accuracy. Moreover, as the elevation angle increases, the variance of elevation angle estimation increases, while the variances of azimuth angle and range estimation decrease. 

## 5. Unambiguous 3-D Localization Estimation

Measurements of phase differences can only be made modulo 2π, which leads to an ambiguity in determining the localization of a source. When $\rho_{0}>\lambda /2$, the phase range can exceed 2π, and phase ambiguity occurs. For the sake of ambiguity resolution, low accuracy angle estimation was first processed, and then the coarse angle estimation was utilized as a reference for accurate angle estimation. Though high-order phase difference is one way to resolve ambiguity, it suffers from estimation accuracy loss [6]. Compression of phase range is another effective way to resolve phase ambiguity. As regards with a UCA, high-order phase differences between adjacent elements associated with the centro-symmetry of a UCA give the same estimation results as the original phases, as well as a compressed phase range. The compressed phase range can be thereafter applied to the phase ambiguity resolution.

### 5.1. Phase Range Compression

According to (4), the range of the phase distribution is determined by the coefficients of the cosine term and the cosine square term. Therefore, reducing their coefficients can compress the phase range. Let us define the phase difference between two adjacent elements, namely

(35) $$\Delta \Phi_{i}=\left\{ \begin{array}{l} \Phi_{i}-\Phi_{i-1},\;i=2,\ldots ,N\\ \Phi_{i}-\Phi_{N},\;i=1 \end{array}\right.$$

Replacing (4) into (35) and neglecting the constant term independent of φ give

(36) $$\begin{array}{ll} \Delta \Phi_{i} & =k\rho_{0}\sin\theta_{s}c_{comp1}\cos\left(\varphi_{i}-\varphi_{s}+\pi /2+\Delta \varphi /2\right)\\ & +k\rho_{0}^{2}/\left(2r_{s}\right)\sin^{2}\theta_{s}c_{comp2}\cos^{2}\left(\varphi_{i}-\varphi_{s}+\pi /2+\Delta \varphi /2\right) \end{array}$$

where

(37) $$c_{comp1}=2\sin\left(\Delta \varphi /2\right)$$

(38) $$c_{comp2}=2\sin\Delta \varphi$$

are the compression coefficients of the cosine term and the cosine square term, respectively. Careful examination of (36) shows that the first-order phase difference leads to the coefficient of the cosine term scaled by 2sin(Δφ/2) and a rotating angle of −(Δφ/2+π/2) from the original array. Further investigation of the coefficient reveals that when Δφ<π/3, or N>6, 2sin(Δφ/2)<1, and the phase range is compressed by a factor of 2sin(Δφ/2). As for the cosine square term, it is scaled by 2sin(Δφ) and rotated by −(Δφ/2+π/2). When N>12, the scale factor is less than 1. These two compression coefficients indicate that the phase range can be compressed by phase differences.

Furthermore, let us define the second-order phase difference, namely

(39) $$\Delta^{2}\Phi_{i}=\left\{ \begin{array}{l} \Delta \Phi_{i+1}-\Delta \Phi_{i},\;i=1,\ldots ,N-1\\ \Delta \Phi_{1}-\Delta \Phi_{i},\;i=N \end{array}\right.$$

Replacing (4) into (39) and neglecting constant terms lead to

(40) $$\Delta^{2}\Phi_{i}=-k\rho_{0}\sin\theta_{s}c_{comp1}^{2}\cos\left(\varphi_{i}-\varphi_{s}\right)-k\rho_{0}^{2}/\left(2r_{s}\right)\sin^{2}\theta_{s}c_{comp2}^{2}\cos^{2}\left(\varphi_{i}-\varphi_{s}\right)$$

**Figure 2:**
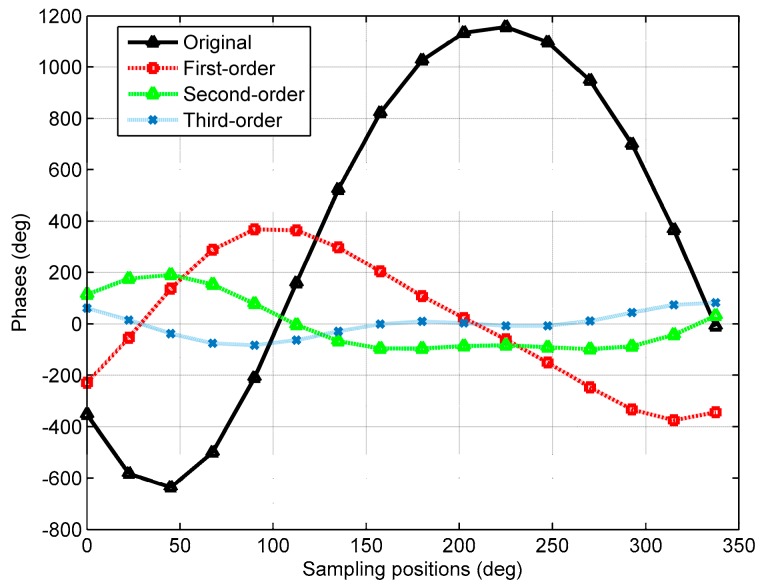
Original and high-order difference phases for *N* = 16, $\rho_{0}=5\lambda$, $\theta_{s}=30{}^{\circ}$, $\varphi_{s}=40{}^{\circ}$, and $r_{s}=6\lambda$.

Recursive manipulations yield the *p*th-order phase difference, namely

(41) $$\begin{array}{ll} \Delta^{p}\Phi_{i} & =k\rho_{0}\sin\theta_{s}c_{comp1}^{p}\cos\left(\varphi_{i}-\varphi_{s}+p\pi /2+h\Delta \varphi /2\right)\\ & +k\rho_{0}^{2}/\left(2r_{s}\right)\sin^{2}\theta_{s}c_{comp2}^{p}\cos^{2}\left(\varphi_{i}-\varphi_{s}+p\pi /2+h\Delta \varphi /2\right) \end{array}$$

where *h* is 1 when *p* is odd and 0 when *p* is even. It is worth pointing out that by simply preprocessing the phase samples, e.g., additions and subtractions, the phase range can be compressed and kept similar to (4) at the same time. Therefore, localization parameters can also be estimated by manipulations of DFT spectrums based on high-order phase differences. The distributions of the original phases and three high-order phase differences are depicted in Figure 2 for *N* = 16, $\rho_{0}=5\lambda$, $\theta_{s}=30{}^{\circ}$, $\varphi_{s}=40{}^{\circ}$, and $r_{s}=6\lambda$. As seen from Figure 2, the phase ranges of the original, first-order, second-order, and third-order phase differences are 1793.2°, 744.1°, 288.2°, 165°, respectively.

### 5.2. HODI of UCA

High-order phase difference leads to similar formulations to the original phase distribution in addition to compression coefficients and rotation angles. Hence the spectrums associated with the localization parameters can be evaluated as

(42) $${\tilde{d}}_{1}^{p}=d_{1}^{p}+\delta d_{1}^{p}$$

(43) $${\tilde{d}}_{2}^{p}=d_{2}^{p}+\delta d_{2}^{p}$$

where

(44) $$d_{1}^{p}=\frac{2\pi }{N}\sum_{i=1}^{N}\Delta^{p}\Phi_{i}\exp\left(j\varphi_{i}\right)=\left(1/2\right)k\rho_{0}{\left(-jc_{comp1}\right)}^{p}\sin\theta_{s}\exp\left(j\varphi_{s}\right)\exp\left(-jh\Delta \varphi /2\right)$$

(45) $$d_{2}^{p}=\frac{2\pi }{N}\sum_{i=1}^{N}\Delta^{p}\Phi_{i}\exp\left(j2\varphi_{i}\right)=k\rho_{0}^{2}{\left(-jc_{comp2}\right)}^{p}\sin^{2}\theta_{s}\exp\left(j2\varphi_{s}\right)\exp\left(-jh\Delta \varphi \right)/\left(8r_{s}\right)$$

are the first-order and the second-order DFT spectrums of the *p*th-order difference of accurate phases, respectively, and

(46) $$\delta d_{1}^{p}=\sum_{i=1}^{N}\Delta^{p}\varepsilon_{i}\exp\left(j\varphi_{i}\right)$$

(47) $$\delta d_{2}^{p}=\sum_{i=1}^{N}\Delta^{p}\varepsilon_{i}\exp\left(j2\varphi_{i}\right)$$

are the first-order and the second-order DFT spectrums of the *p*th-order difference of noises, respectively. Compensating the compression coefficients and neglecting noises, the three localization parameters of the source are readily calculated as counterparts to (24)–(26), respectively, namely

(48) $${\tilde{\theta }}_{s}^{p}=\sin^{-1}\left(2\left|{\left(-jc_{comp1}\right)}^{-p}\exp\left(jh\Delta \varphi /2\right){\tilde{d}}_{1}^{p}\right|/\left(Nk\rho_{0}\right)\right)$$

(49) $${\tilde{\varphi }}_{s}^{p}=\arg\left({\left(-jc_{comp1}\right)}^{-p}\exp\left(jh\Delta \varphi /2\right){\tilde{d}}_{1}^{p}\right)$$

(50) $${\tilde{r}}_{s}^{p}=\left|{\left({\left(-jc_{comp1}\right)}^{-p}\exp\left(jh\Delta \varphi /2\right){\tilde{d}}_{1}^{p}\right)}^{2}/\left(2k{\tilde{d}}_{2}^{p}{\left(-jc_{comp2}\right)}^{-p}\exp\left(jh\Delta \varphi \right)\right)\right|$$

The two angular parameters are hence extracted from the first-order DFT spectrum of high-order phase differences, and the range parameter is manipulated from the first-order and the second-order DFT spectrums of high-order phase differences as well. 

By comparisons of (44) to (18) and (45) to (19), respectively, the two DFT spectrums of the *p*th-order phase difference of accurate phases become

(51) $$d_{1}^{p}={\left(-jc_{comp1}\right)}^{p}\exp\left(-jh\Delta \varphi /2\right)d_{1}$$

(52) $$d_{2}^{p}={\left(-jc_{comp2}\right)}^{p}\exp\left(-jh\Delta \varphi \right)d_{2}$$

Meanwhile, the two DFT spectrums of the *p*th-order difference of phase noises lead to

(53) $$\delta d_{1}^{p}={\left(-jc_{comp1}\right)}^{p}\exp\left(-jh\Delta \varphi /2\right)\delta d_{1}$$

(54) $$\delta d_{2}^{p}={\left(-jc_{comp2}\right)}^{p}\exp\left(-jh\Delta \varphi \right)\delta d_{2}$$

As seen from (51) to (54) that the spectrums of accurate phases and phase noises are compressed to the same extent, i.e., ${\left(-jc_{comp1}\right)}^{p}\exp\left(-jh\Delta \varphi /2\right)$ for the first-order DFT spectrum and ${\left(-jc_{comp2}\right)}^{p}\exp\left(-jh\Delta \varphi \right)$ for the second-order DFT spectrum. Hence the localization parameters, i.e., the elevation angle, the azimuth angle, and the range, calculated by (24)–(26) using ${\tilde{\Phi }}_{i}$ are exactly the same as those by (48)–(50) using $\Delta^{p}{\tilde{\Phi }}_{i}$, respectively. This indicates that calculations of localization parameters using high-order phase differences give invariant results as the original phase measurements. Therefore, this method is termed high-order difference invariance, or HODI for short. As a result, we can safely adopt the high-order phase differences to estimate localization parameters without accuracy loss. Note that the parameter estimation and the ambiguity resolution are processed simultaneously, only with several addition and subtraction operations of the phase data. The ambiguity resolution and the parameter estimation are merged into a single process, since no coarse parameter estimations are required.

Concerning the computational complexity of the proposed estimation algorithm, the extraction of phases needs *MN* complex multiplications and (*M* − 1) × *N* complex additions, as in (5). Ambiguity preprocessing requires *N* real additions, as in (35) or 2*N* real additions, as in (39). The first-order and second-order DFTs require *N* complex multiplications and (*N* − 1) complex additions, as in (46) and (47), respectively.

## 6. Simulation Results

**Figure 3:**
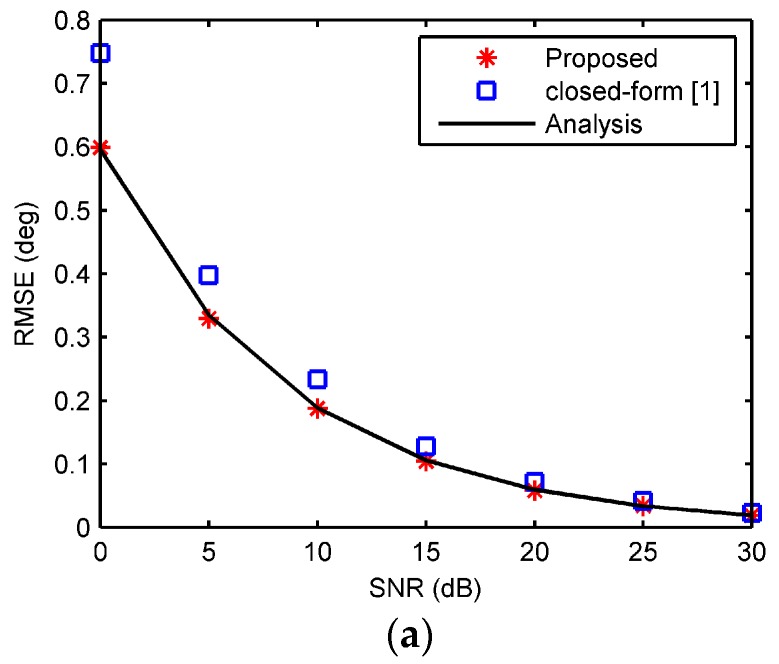

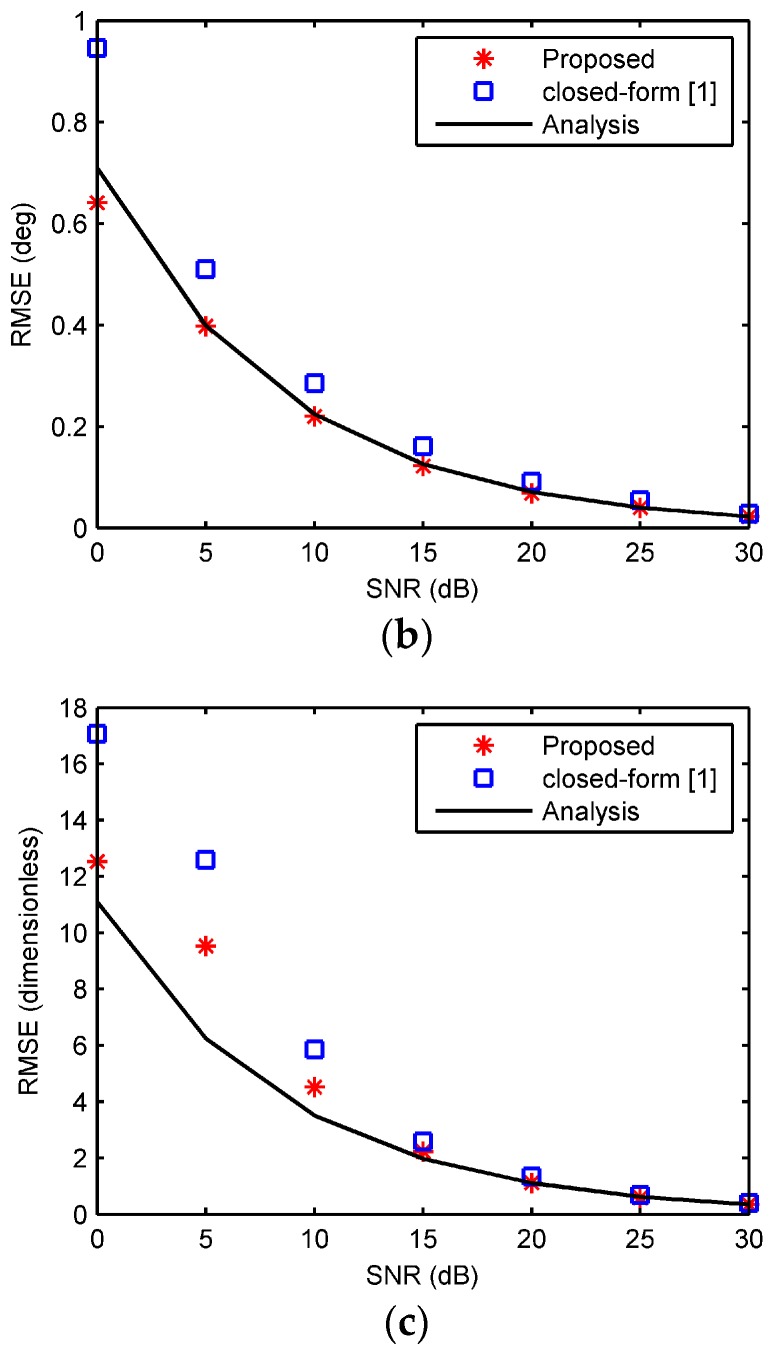
RMSE against signal-to-noise ratios (SNRs). (**a**) The elevation angle; (**b**) the azimuth angle; (**c**) the range.

In order to compare with the closed-form algorithm [1], assuming only sampling noises exist, a UCA consisting of 16 elements with a radius of $\rho_{0}=0.25\lambda$ is exemplified. The number of snapshots was 200. In the first example, phase noises were considered as AWGN with SNR from 0 dB to 30 dB. 500 Monte Carlo simulations were run for calculating 3-D localization parameters employing (18)–(21). The root mean square errors (RMSEs) of elevation angle, azimuth angle, and range were estimated for performance evaluation. Note that the RMSE of range estimation was normalized by the array radius. The source was fixed at $\left(\theta_{s},\varphi_{s},r_{s}\right)=\left(35^{\circ },120^{\circ },3\lambda \right)$. The performance of the proposed algorithm against SNRs is shown in Figure 3. The estimations of the closed-form algorithm [1] with *l* = 3 and the analysis results described in (31), (32), and (34) are also shown for comparison. As shown in Figure 3, the RMSEs of 3-D localization estimations agree with the analysis in Section 4. The RMSEs by [1] are larger than the proposed algorithm and hence less accurate, in that the LSE in [1] has fewer terms than the number of sensors. 

**Figure 4:**
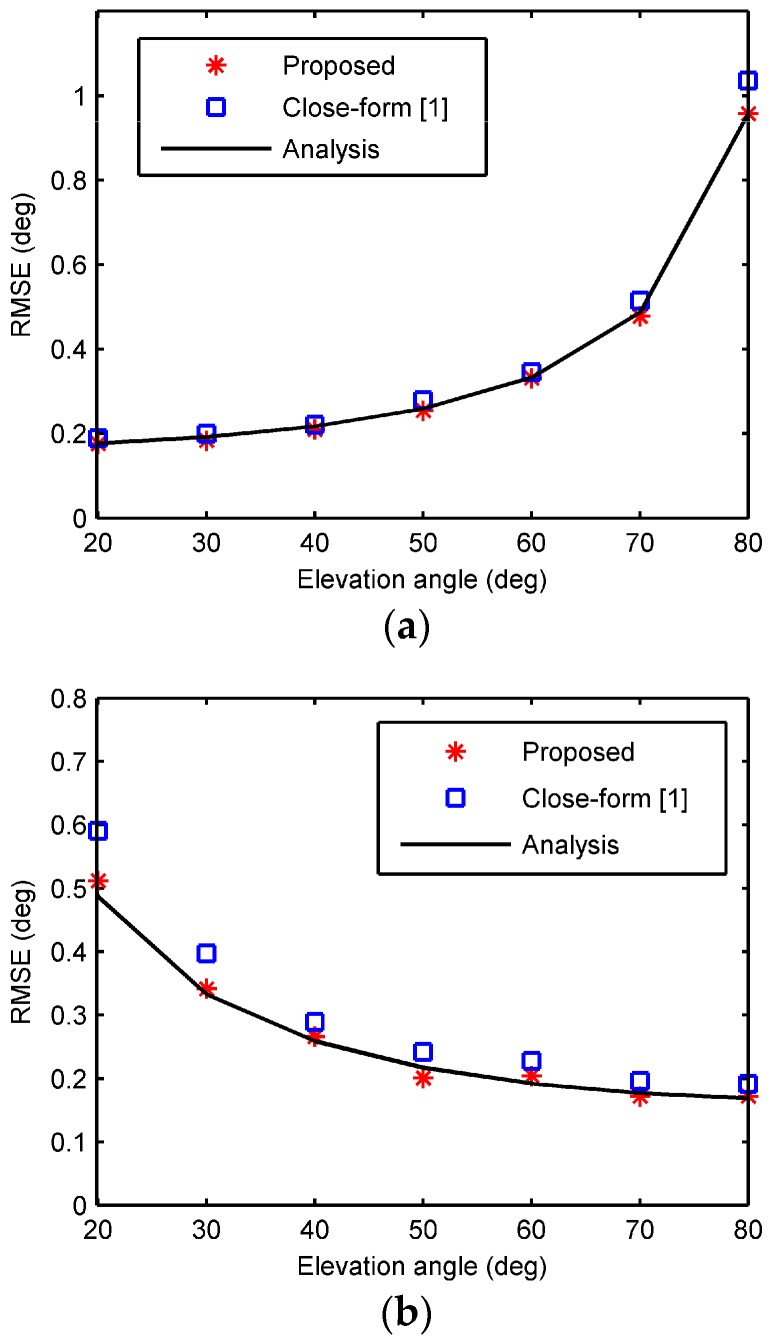

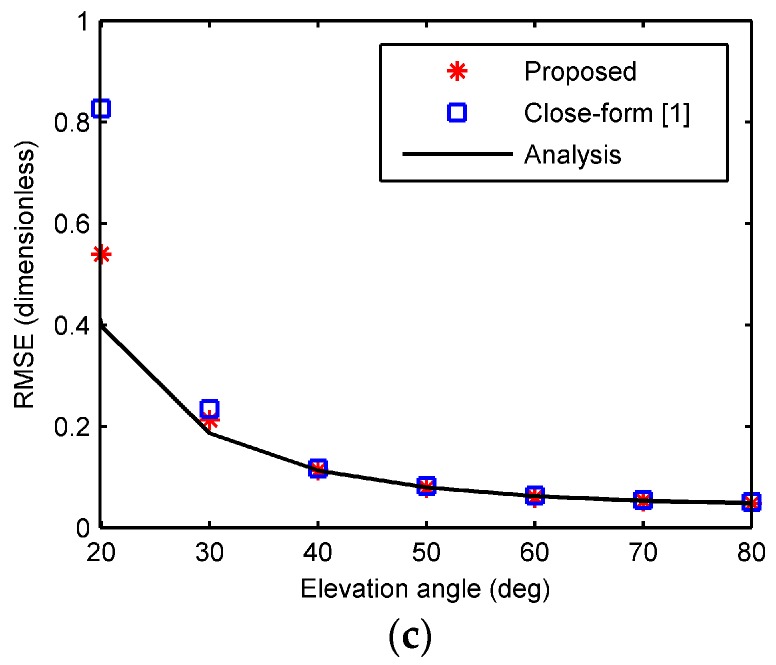
RMSE against elevation angles. (**a**) The elevation angle; (**b**) the azimuth angle; (**c**) the range.

The second example investigated the estimation accuracy against elevation angles. A Rayleigh fading channel [18] was assumed that the real and imaginary parts of the response are modeled by independent and normally distributed zero-mean processes. The SNR was 10 dB, and the azimuth angle was fixed at 10°, while the elevation angle varied from 20° to 80°. The calculated RMSEs are plotted in Figure 4, as well as those by the closed-form algorithm and the analytical results. As seen, the simulated results of the proposed algorithm are in agreement with the predictions and more accurate than the closed-form algorithm. Meanwhile, the elevation angle estimation accuracy decreases with the incident elevation angle, while the estimation accuracies of the azimuth angle and the range increase with the incident elevation angle. 

## 7. Experimental Results

**Figure 5:**
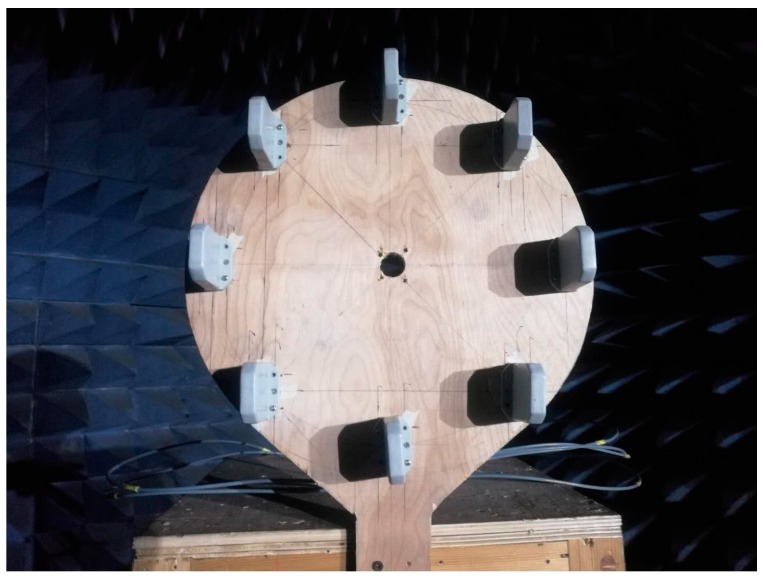
Measured UCA with a radius 0.15 m and 8 log periodic dipole antennas.

To validate the effectiveness of the proposed algorithm, a UCA with a radius of $\rho_{0}$ = 0.15 m consisting of 8 log periodic dipole antennas was utilized for an experiment in a microwave anechoic chamber, as illustrated in Figure 5. The UCA was placed on a rotator, whose rotating axis aligned with the center of the UCA in a vertical line, scanning the incident angle horizontally from −45° to 45°. The distance between the transmitting antenna and the center of the receiving array was 0.44 m. The operating frequency was 2.6 GHz. A network analyzer was used to measure the phase of each antenna element. The SNR is 30 dB and phase measurement noises mainly arose from hardware imperfections.

**Figure 6:**
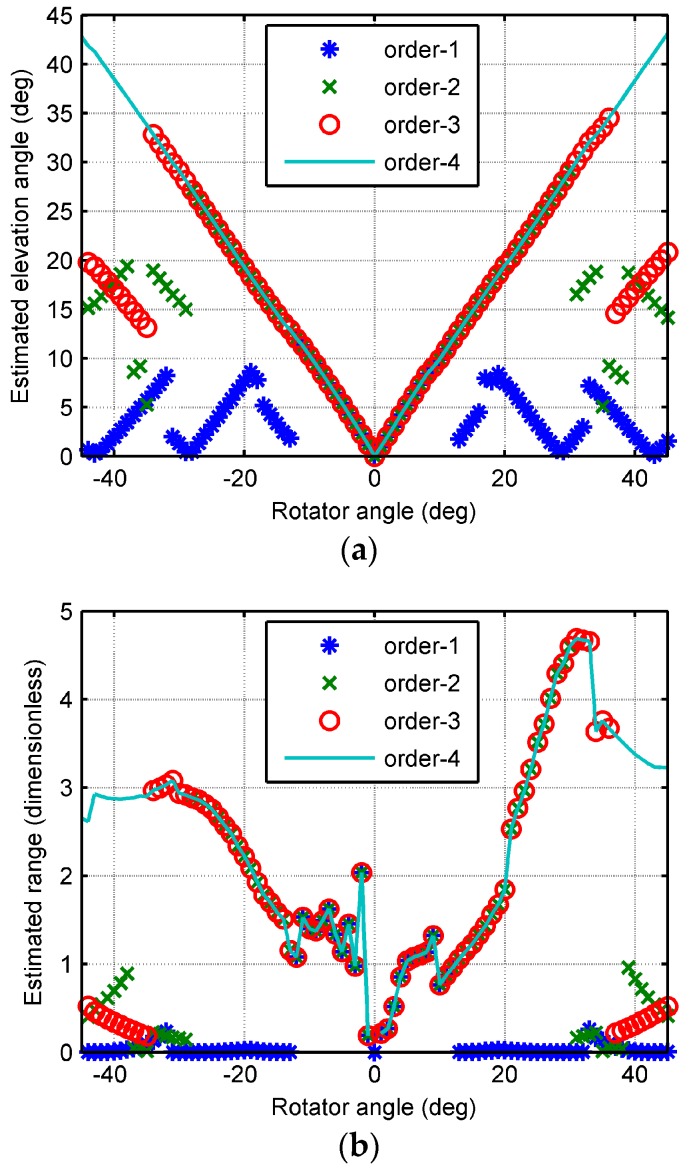
Estimated results by variational phase difference orders. (**a**) The elevation angle; (**b**) the range.

Since the motion of the rotator scanned in only one dimension, the elevation angle and the range were evaluated. The estimated elevation angle and range by phase difference of first order, second order, third order, and fourth order are shown in Figure 6. As seen from Figure 6, phase ambiguity was successfully resolved by the fourth-order phase difference. However, phase ambiguity resolution failed when the absolute value of the incident angle is more than 21°, 30°, and 36°, adopting first-order, second-order, and third-order phase differences, respectively. The reason is that the maximum unambiguous angle is the elevation angle corresponding the phase range that does not exceed 2π. The maximum unambiguous elevation angles with respect to first-order, second-order, and third-order phase difference are 31°, 42°, and 53°, respectively. Taking phase noises into account, the successful resolved angle is smaller than the maximum unambiguous angle. 

**Figure 7:**
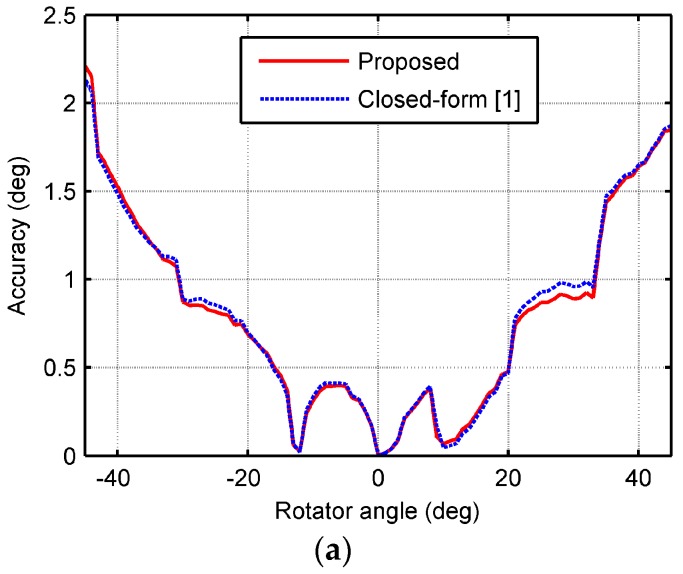

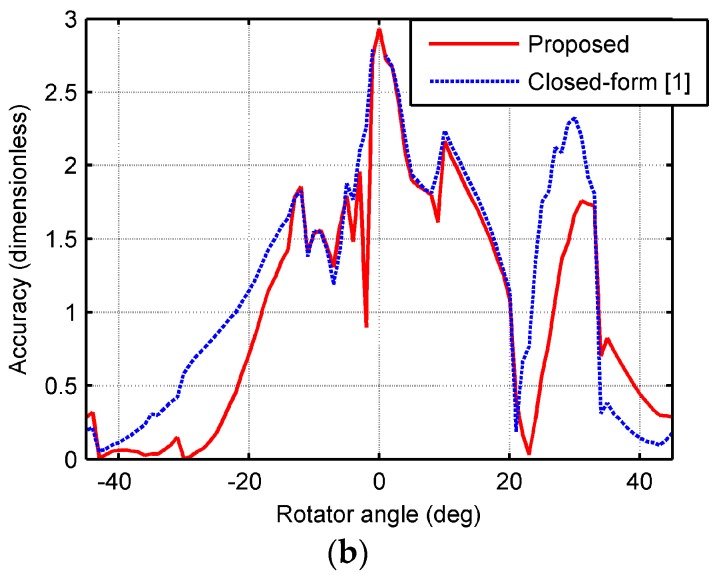
Estimated accuracies comparison of the proposed algorithm and the closed-form algorithm. (**a**) The elevation angle; (**b**) the range.

The successfully resolved elevation angle and range are shown in Figure 7 with those by the closed-form algorithm [1] for comparison. As seen from Figure 7, the proposed algorithm performed better than the closed-form algorithm, since the estimation accuracies were generally higher than the closed-form algorithm.

## 8. Conclusions

This paper has presented an analytic and unambiguous algorithm for phase-based 3-D localization estimation of a single source with a UCA. Analytic and explicit formulae for 3-D position parameters have been derived by exploiting Fourier transforms. 3-D parameters are decoupled to two spectrums in the Fourier domain. Algebraic relations have been established between the 3-D parameters and the DFT spectrums of discrete phase samples. The proposed algorithm has sufficiently exploited the centro-symmetry and periodicity of circular arrays. Fourier sampling theorem ensures that at least five sensors can obtain 3-D position parameters of a single source using a UCA. Accuracy analysis in the Fourier domain has provided mathematical insights into advantages of the localization estimation algorithm. Furthermore, the high-order difference invariance (HODI) property of a UCA has been addressed and exploited to resolve phase ambiguity. Therefore, a major advantage of the proposed algorithm is that ambiguity resolution and 3-D localization are both explicit and are processed simultaneously. Finally, simulations and experiments have validated the expected benefits of the proposed analytic and unambiguous 3-D localization algorithm with a UCA. Future work may focus on studies of array configurations to improve the range estimation accuracy near the normal direction of a UCA.
